# Day-to-day associations between mindfulness and perceived stress: insights from random intercept cross-lagged panel modeling

**DOI:** 10.3389/fpsyg.2024.1272720

**Published:** 2024-04-16

**Authors:** Olaf Borghi, Martin Voracek, Ulrich S. Tran

**Affiliations:** ^1^Department of Cognition, Emotion, and Methods in Psychology, Faculty of Psychology, University of Vienna, Vienna, Austria; ^2^University Research Platform “The Stress of Life (SOLE) – Processes and Mechanisms underlying Everyday Life Stress”, University of Vienna, Vienna, Austria

**Keywords:** mindfulness, daily hassles, stress, random intercept cross-lagged panel model (RI-CLPM), longitudinal, structural equation modeling (SEM), daily diary study

## Abstract

**Objective:**

Mindfulness is frequently seen as a protective factor of stress, but self-report measures of mindfulness may overlap with other related constructs, such as mental health, and could thus not only be a predictor, but also an outcome of stress. This study thus aimed to examine the longitudinal bidirectional associations between the use and perceived helpfulness of the four mindfulness facets Observe, Describe, Nonjudge, and Nonreact with daily perceived stress.

**Methods:**

Participants from a large (*N* = 1,276) mixed student and community group sample filled out a brief daily diary over the time span of 7 days. Bidirectional cross-lagged effects were investigated using the random-intercept cross-lagged panel model, an extension of the traditional cross-lagged panel model that allows to differentiate between stable between-unit differences and time-varying within-unit dynamics. In addition, we controlled for several baseline and sociodemographic confounders.

**Results:**

At the within-subject level, the use of Actaware was associated with higher perceived stress on the next day (*β* = 0.03, *p* = 0.029). The use (*β* = −0.04, *p* = 0.025) and perceived helpfulness (*β* = −0.05, *p* = 0.014) of Nonreact were associated with lower perceived stress on the next day. In turn, perceived stress was associated with lower perceived helpfulness of Describe (*β* = −0.04, *p* = 0.037) and Nonreact (*β* = −0.03, *p* = 0.038) on the next day. In addition, there were several residual correlations between mindfulness facets and perceived stress within days. At the between-subject level, there was a positive association between the random intercept of Describe and daily stress (*r* = 0.15, *p* = 0.003). In addition, while baseline perceived stress was negatively associated with the random intercepts of the mindfulness facets, two baseline components of mindfulness were not associated with the random intercept of perceived stress.

**Conclusion:**

On the currently investigated time scale, our results challenge prior results and assumptions regarding mindfulness as a buffering and protective factor against daily stress. With the exception of Nonreact, mindfulness was either positively associated with perceived stress, or in turn perceived stress appeared to interfere with the ability to stay mindful in daily life.

## 1 Introduction

When the demands of our environment exceed our adaptive capacities, we experience stress (Cohen et al., 2007). Already minor daily hassles, such as arguing with friends or family, can be experienced as stressful, and contribute to overall levels of stress (Chamberlain and Zika, 1990; Almeida, 2005; Donald et al., 2016). Therefore, daily stressors can both have an immediate impact on our personal well-being, but also accumulate and lead to more serious negative reactions (Almeida, 2005), and in the long run negatively influence our physical and mental health (DeLongis et al., 1988; Serido et al., 2004).

However, individuals vary in their responses to stressful events, and adaptive personal and social resources may allow us to cope better with them (Almeida, 2005). Protective factors that can buffer the negative implications of stressful events in our everyday lives are thus highly relevant (Wu et al., 2013). As such, mindfulness programs, such as mindfulness-based stress reduction (MBSR; Kabat-Zinn, 1982), have been reported to reduce stress and increase quality of life (Khoury et al., 2015). Mindfulness as a personality trait was associated with more adaptive stress-responses and lower levels of perceived stress as well (Weinstein et al., 2009; Bao et al., 2015). This stress-buffering account of mindfulness was suggested as a central pathway between associations of mindfulness and health, both through bottom-up (i.e., lower stress-reactivity) and top-down processes (i.e., adaptive emotion regulation in the face of stress; Creswell and Lindsay, 2014). Together, such findings caused considerable attention to mindfulness as a potential protective factor that could reduce negative health outcomes associated with stress (Conner and White, 2014; de Frias and Whyne, 2015; Conversano et al., 2020).

Originally, the concept of mindfulness has its roots in Buddhist meditation practices (Kabat-Zinn, 1994, 2003; Bodhi, 2011). It was introduced in Western psychology and medicine in the second half of the 20th century and quickly gained popularity (Kabat-Zinn, 2013). One of the most commonly referred definitions describes mindfulness as purposeful and non-judgmental moment-to-moment awareness (Kabat-Zinn, 1994, p. 4; Kabat-Zinn, 2003). In another influential operational definition, Bishop et al. (2004) proposed two components of mindfulness, self-regulated attention (SRA) and orientation to experience (OTE). SRA describes the attentional component of mindfulness and the ability to intentionally bring one’s attention to the present moment. OTE describes an open, accepting, and non-judgmental attitude toward one’s own experiences and the present world (Bishop et al., 2004). Other approaches define mindfulness as a collection of related processes including acceptance, defusion, contact with the present moment, and a transcendent sense of self (Fletcher and Hayes, 2005). Common mindfulness skills (such as observing and describing) as well as the way how these skills should be performed (such as non-judgmentally, accepting, in the present moment, and effectively) were suggested as well (Dimidjian and Linehan, 2003).

Many of these aspects of mindfulness are assessed in widely-used mindfulness self-report inventories, such as the Five Facet Mindfulness Questionnaire (FFMQ, Baer et al., 2006). As a prominent measure of trait mindfulness, the FFMQ assesses mindfulness with five facets (i.e., scales) Observe (actively perceiving internal and external stimuli), Describe (the ability and tendency to describe internal experiences with words), Actaware (being attentive toward one’s own actions), Non-judge (taking a neutral, non-judgmental stance toward one’ own thoughts and feelings) and Non-react (attending to feelings and thoughts without being carried away by them). Interestingly, these facets could possibly also be subsumed under the two-component model of mindfulness (Bishop et al., 2004). Actaware and Non-judge were suggested to load on OTE and Observe on SRA. Describe and Non-react were suggested to load on both factors (Tran et al., 2013). This two-dimensional structure of the FFMQ yielded good model fit across multiple studies (Tran et al., 2013, 2014; Burzler et al., 2019; Borghi et al., 2023), and combines the advantages of the empirically derived five-facetted structure of the FFMQ with the theoretical two-component model of mindfulness.

The general tendency to be mindful (trait mindfulness) can be distinguished from an individual’s degree of mindfulness at a specific time point (state mindfulness; Medvedev et al., 2017). Increases in state mindfulness may lead to increases in trait mindfulness (Kiken et al., 2015), but increases in trait mindfulness may also lead to increases in state mindfulness [see Burzler and Tran (2022)]. Thus, effects of state and trait mindfulness can be hard to disentangle (Medvedev et al., 2017), and this can lead to difficulties when studying mindfulness only at single or only a few time points [for a further discussion of this topic and also on the relevant differentiation of trait mindfulness into dispositional and cultivated aspects in this context, see Burzler and Tran (2022)].

Adding to this, there is currently no scientific consensus on the exact definition of mindfulness (Van Dam et al., 2018), and especially self-reported mindfulness has been criticized in regard to its construct validity and conceptual ambiguity (Goldberg et al., 2017). Accordingly, the results of a recent meta-analysis indicated that self-reported mindfulness is no unique mediator of the effects of mindfulness interventions, but instead may (at least partially) be rather a correlate or consequence of self-reported mental health (Tran et al., 2022). Psychometric findings indicate that common factors underlie the five facets of mindfulness and proposed mechanisms of mindfulness (e.g., emotion regulation, attention regulation; Bednar et al., 2020). Thus, self-report measures of mindfulness, such as the FFMQ, may not only measure mindfulness, but also some of its supporting mechanisms. This could explain increases in trait mindfulness following non-mindfulness-based therapies (e.g., Tran et al., 2022) and the overlap of trait mindfulness with other constructs, such as neuroticism, emotion regulation, and mental health [see Tran et al. (2020)]. Yet, these trait overlaps are likely also facilitated by semantic and sentiment similarities of the item contents of the respective widely used scales (Fischer et al., 2023).

Based on these potential trait overlaps of mindfulness with related constructs, one could expect longitudinal bidirectional associations between mindfulness, neuroticism and mental health: And indeed, mindfulness is longitudinally related to lower neuroticism and neuroticism to lower mindfulness (Wang et al., 2022). Also, lower mindfulness does not only predict lower mental health longitudinally, but lower mental health predicts lower mindfulness as well [Kocovski et al., 2015; Gómez-Odriozola and Calvete, 2020; but see Snippe et al. (2015)].

Such findings raise the question of whether mindfulness may have unique effects on perceived stress and whether stress may not also influence day-to-day mindfulness. One previous multi-study investigation Weinstein et al. (2009) reported that mindfulness negatively predicts perceived stress in daily data and is positively associated with the use of adaptive coping mechanisms. Another study Donald et al. (2016) reported specific positive effects of present-moment awareness (corresponding to Actaware in the FFMQ) on more self-efficacious coping and small *positive* associations with perceived daily stress. This indicates that mindfulness may (beneficially) affect coping with stress, but does not necessarily decrease perceived stress levels. However, none of these prior studies investigated the possibility of bidirectional effects. Stress was treated only as an outcome in analysis.

Given this prior evidence, a re-investigation of both the associations of mindfulness facets with perceived stress and the clarification of the temporal order of effects (i.e., from perceived stress to mindfulness facets or vice versa) remains an important research desiderate. In this context, a methodologically interesting approach is the random intercept cross-lagged panel model (RI-CLPM; Hamaker et al., 2015), an extension of the traditional cross-lagged panel model (CPLM). The RI-CLPM allows investigating longitudinal cross-lagged paths (thereby testing for the possible bidirectionality of associations), while simultaneously differentiating between trait-like between-subject components and state-like within-subject fluctuations. While this analytic approach has been recently used to investigate the longitudinal associations between mindfulness and neuroticism (Wang et al., 2022), to our knowledge the RI-CLPM has not yet been applied to investigate the longitudinal associations between mindfulness and perceived stress.

### 1.1 The present research

In summary, mindfulness is frequently seen as a protective factor of stress and previous studies indeed indicated longitudinal associations of mindfulness on responses to stressors in the everyday life (Weinstein et al., 2009; Donald et al., 2016). However, it is possible that the longitudinal associations between mindfulness and stress are bidirectional and may depend on specific mindfulness facets. Further, both daily mindfulness and perceived stress may be affected by confounding variables, such as participants’ meditation experience and trait mindfulness levels and baseline levels of perceived stress. The capability and likelihood of being, and staying, mindful in everyday life likely depends on baseline mindfulness levels and prior experience of cultivating mindfulness skills (Burzler and Tran, 2022). Differences in mindfulness between student and non-student (i.e., community) samples have been reported as well (e.g., Tran et al., 2013). Lastly, perceived stress and mindfulness were both associated with age and biological sex in prior research (e.g., Weinstein et al., 2009).

In the present study, we thus aimed to provide a comprehensive investigation with the three study goals of (1) investigating the longitudinal associations between different mindfulness facets and perceived stress in daily life and (2) clarifying their temporal order, while (3) controlling for a number of baseline and sociodemographic variables: the two components of trait mindfulness, SRA and OTE; meditation experience; baseline perceived stress; student status; and participant sex and age. For this, we examined the associations between the daily use and perceived helpfulness of the mindfulness facets Describe, Actaware, Nonjudge, and Nonreact with perceived stress across the time span of seven subsequent days in a large, mixed student and community sample, utilizing the RI-CPLM. The Observe facet was not assessed, based on prior findings of weak or no associations of Observe with positive mental health and stress-relieving outcomes (e.g., Bergomi et al., 2013; Medvedev et al., 2018). The traditional CPLM has been applied in the past in the investigation of the longitudinal bidirectional associations between mindfulness and mental-health-related outcomes (e.g., Gómez-Odriozola and Calvete, 2020; Tumminia et al., 2020). However, this approach fails to account for person-level associations and potentially leads to spurious cross-lagged effects or the underestimation of true effects (Lucas, 2023). To investigate whether the application of the simpler CPLM would have led to biased results, we also provide results with this model for comparison.

**Figure 1:**
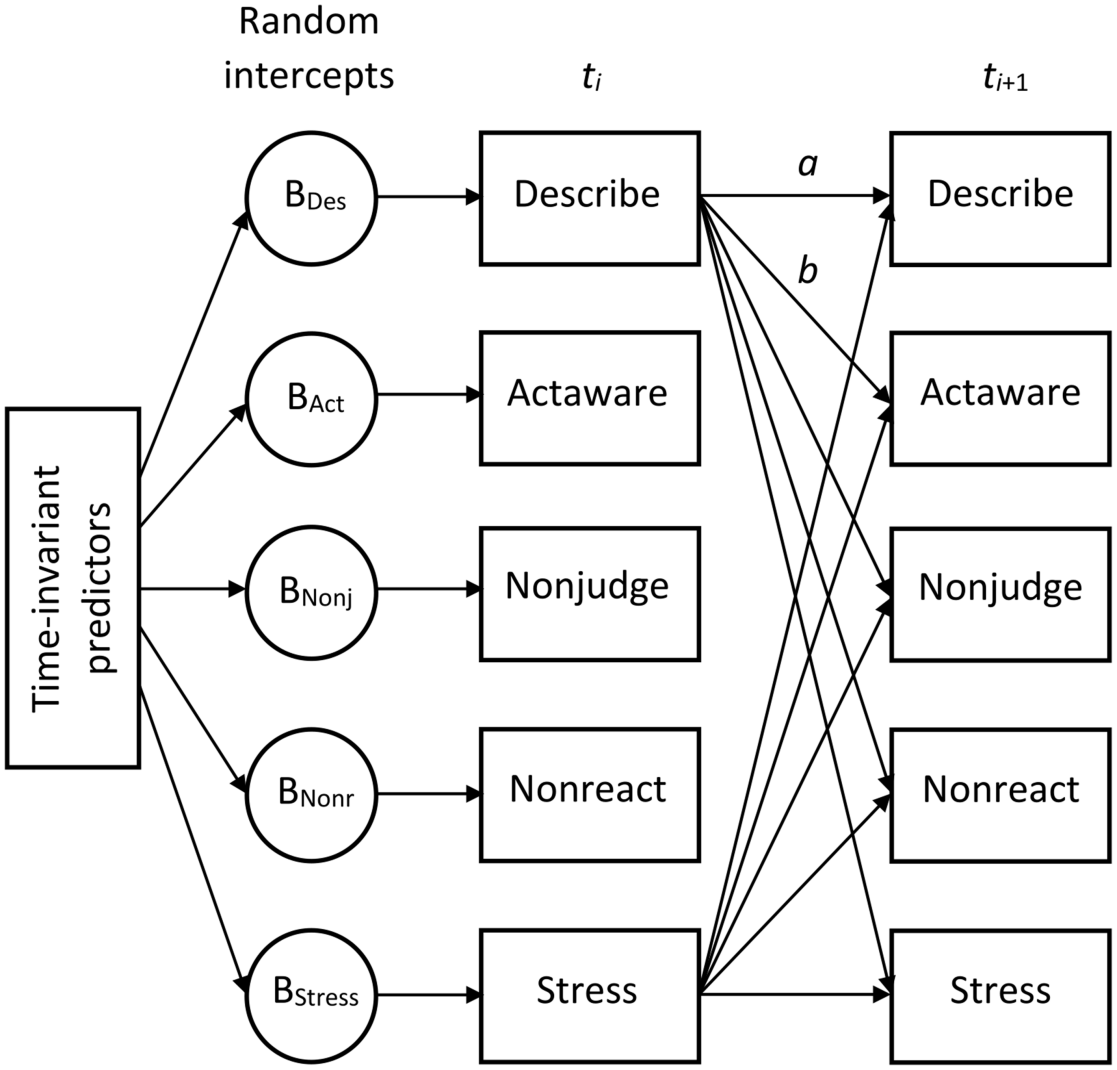
Simplified schematic representation of the statistical models. *t_i_*, time point (with *i* going from 1 to 6); *a*, autoregressive effect; *b*, cross-lagged effect. The actual models included autoregressive effects for all variables and cross-lagged effects between all variables. The residuals of all daily measured variables were allowed to intercorrelate at each time point (t2–t7; for t1, the variables were allowed to covary), as were the residuals of all random intercepts, which captured the trait-like between-subject differences of the trajectories of the individual participants. The random intercepts had paths to their associated variables at each time point. All time-invariant predictors were allowed to intercorrelate. In Models 2 and 3, they had paths to each of the random intercepts (as depicted). In Models 1 and 4, the time-invariant predictors had instead paths to each daily measured variable. The structure of the within-subject part of the models is presented in a simplified form. The actual models also contained one latent variable per daily measured variable and autoregressive and cross-lagged effects were between these latent variables, not between the observed variables (as depicted).

For the within-subject level, we expected that everyday use and perceived helpfulness of mindfulness facets predict lower daily stress longitudinally (H1), but that daily stress longitudinally predicts the everyday use and perceived helpfulness of mindfulness facets as well (H2). At the between-subject level and across the investigation period (seven consecutive days), we expected that the overall higher use or perceived helpfulness of the mindfulness facets is associated with overall less perceived stress (H3). A schematic display of the hypothesized model is provided in Figure 1.

## 2 Materials and methods

### 2.1 Participants

This study used data from a mixed student and community sample, with a total of *N* = 1,276 German-speaking participants (53% women; age: *M* = 32.4, *SD* = 14.7 years, range: 17–85 years). Sample characteristics are displayed in more detail in Table 1.

**Table 1:** Sample characteristics.

Characteristic	*n*	%
Sex		
Female	671	52.6
Male	602	47.2
Not specified	3	0.2
Nationality		
Austria	633	49.6
Germany	566	44.4
Other/not specified	77	6
Highest educational level		
Compulsory/vocational education	233	18.3
Upper secondary education	706	55.3
Tertiary education	324	25.4
Not specified	13	1.01
Currently studying	636	49.8
Currently employed	753	59.0
Meditation frequency		
Never/not regularly	1,075	84.3
At least once a week	166	13.0
Not specified	35	2.7
Most common types of meditation practice^a^		
Yoga	84	50.6
Zen	17	10.2
Other	65	39.2

Total *N* = 1,276 German speaking adults. ^a^Among all participants who meditated at least once a week (*n* = 166).

### 2.2 Procedure

Participants were contacted by 22 research assistants through word-of-mouth advertising and were invited to fill out a printed questionnaire or an electronic PDF. The questionnaire alongside an informed consent form was either personally handed out to participants or sent via email. Participants were asked to provide information on their sociodemographic background and meditation experience. As the data were assembled within a larger research project, participants filled out a total of eight self-report instruments, of which two were relevant for this study (for an overview of all administered scales as well as their respective position within the survey, see https://osf.io/7tnwq/).

In addition, participants were asked to fill out a brief diary over the time span of 7 days at the end of the questionnaire, assessing the use and perceived helpfulness of four mindfulness skills, as well as perceived daily stress levels, at the end of each day. Afterwards, the questionnaires were again personally collected by the research assistants or obtained via email.

Participation was completely voluntary and all data were fully anonymous. No identifying personal information was collected in the questionnaire (also, the consent form was not signed by name, but ticked for agreement, like it is customary in online surveys; emails were deleted after saving the attached and filled-out PDFs with generic and non-identifying file names) and all data were entered and made available in the database only by persons not in direct contact with the participants. All data collection took place prior to the COVID-19 pandemic.

### 2.3 Measures

All scales and items were presented in German. For all scales, McDonald’s ω and Cronbach’s *α* were calculated using the MBESS package in R (Kelley, 2022). Sample reliabilities for all scales were >0.70 and are displayed in Supplementary Table S1.

#### 2.3.1 Five facet mindfulness questionnaire

Trait-mindfulness was assessed using the German 23-item short form (Tran et al., 2013) of the Five Facet Mindfulness Questionnaire (FFMQ; Baer et al., 2006). The facets Observe, Describe and Nonjudge were measured by four items, and Nonreact was measured with all seven items of the full form. All items were rated on 5-point Likert scales ranging from 1 = *never or very rarely true* to 5 = *very often or always true*. The full form of the FFMQ showed high reliability and validity across different samples (Baer et al., 2008), and the German short form showed improved psychometric properties compared with the full form (Tran et al., 2013).

#### 2.3.2 Perceived stress questionnaire

Subjectively perceived stress prior to the diary assessment was assessed using a sum score of the German revision of the Perceived Stress Questionnaire (PSQ; Fliege et al., 2005), which comprises 20 items that are rated on a 4-point Likert scale (1 = *almost never*; 2 = *sometimes*; 3 = *often*; 4 = *usually*). The German short form of the PSQ showed high reliability and high validity (Fliege et al., 2005).

#### 2.3.3 Meditation experience

Different aspects of meditation experience were assessed with several items. The first item assessed the subjectively perceived experience with meditation or mindfulness practices on a 5 point-Likert scale ranging from 1 = *no experience*, 2 = *a little*, 3 = *some*, 4 = *a lot*, to 5 = *very much experience*. The second item targeted the frequency of meditation or mindfulness practice (0 = *never*; 1 = *not regularly*; 2 = *once a week*; 3: *twice a week*; 4 = *three times a week*; 5 = *four times a week or more*). In addition, participants were asked to provide information on their average meditation/mindfulness practice duration on a day in minutes, as well as the total years of practice. Lastly, participants also reported their most practiced meditation type. While most of these items served for mere descriptive information, based on previous studies, the frequency of meditation or mindfulness practice was included as an indicator of meditation experience in the analysis of this study (Soler et al., 2014; Cebolla et al., 2017).

#### 2.3.4 Daily diary measures

A short survey consisting of five single items was used to assess the use and perceived helpfulness of four mindfulness facets and perceived stress levels across seven time waves (t1–t7) at the end of each of the seven subsequent days (the original German items and English translations can be found on https://osf.io/7tnwq/). Experience sampling studies frequently make use of single-item measures and such single items were also used in a previous study on the effects of present-moment awareness on stress (Donald et al., 2016). Single items are generally seen as a psychometrically appropriate, flexible, and brief alternative that can yield similar results like multi-item measures when administering long or full forms of psychometric self-report measures is infeasible (Wanous et al., 1997; Gardner et al., 1998; Fuchs and Diamantopoulos, 2009; Gogol et al., 2014). In this study, the use of single item measures allowed to enroll a large sample size over seven time waves.

The four mindfulness facets Describe, Actaware, Nonjudge, and Nonreact were assessed with four items that were rated with two response scales each concerning their daily use and perceived helpfulness, all rated on scales ranging from 1 = *not at all* to 10 = *very much*. Item contents were taken and adapted from items of the FFMQ, a self-report scale with high validity and reliability (e.g., Baer et al., 2008; Tran et al., 2013). The perceived stress level on each day was rated with a single item, ranging from 1 = *none* (no stress on the given day) to 10 = *maximum* (highest subjective stress level on the given day).

### 2.4 Statistical analyses

#### 2.4.1 Exploratory structural equation modeling

The two-factor higher-order structure of the five facets of mindfulness was investigated using exploratory structural equation modeling (ESEM; Marsh et al., 2014). ESEM is a combination of exploratory factor analysis (EFA) and confirmatory factor analysis (CFA), in which items are allowed to cross-load on factors, while the same model fit indices can be obtained as in classical CFA. Therefore, ESEM can be seen as an integration of (some) the best features of EFA and CFA, with realist assumptions (i.e., closer to a “messy reality”), while simultaneously preserving the flexible analytic possibilities of CFA (Marsh et al., 2014).

The ESEM has already been applied to derive two higher-order factors of the FFMQ in multiple previous studies (Tran et al., 2013, 2014; Burzler et al., 2019; Borghi et al., 2023). Therefore, in this study, as suggested for cases with a more clearly defined *a-priori* factor structure, target rotation was used (Marsh et al., 2014). Based on theoretical considerations [see Tran et al. (2013)] and empirical results from the previous studies, estimation of the loadings on SRA was free for Observe, Describe and Nonreact, and constrained to zero for Actaware and Nonjudge, whereas for OTE the loadings from Describe, Actaware, Nonjudge and Nonreact were free, while the loading of Observe was constrained to zero (CFA within ESEM analysis). Analyses were performed in R version 4.2.1 using the ESEMcomp package version 0.2 (Silvestrin and De Beer, 2022).

As the factor loadings of the five facets of mindfulness on the two higher-order factors were shown to differ between student and non-student (i.e., community) samples (Tran et al., 2013), a multigroup analysis was performed, with group (0 = community; 1 = student) as the grouping variable. Based on the results from the multigroup ESEM analysis, group-specific regression factor scores were obtained and used in subsequent analysis (DiStefano et al., 2009).

#### 2.4.2 Random intercept cross-lagged panel modeling

To test our main hypotheses, RI-CLPMs were used (Hamaker et al., 2015). The RI-CLPM is an extension of the classic cross-lagged panel model which specifically and additionally models cross-lagged relationships and autoregressive effects of variables over time. In contrast to the traditional approach, the RI-CPLM also allows differentiating between stable between-unit differences and time-varying within-unit dynamics. Longitudinally observed variables are decomposed into several components: A grand mean for each variable across all time waves; random intercepts capturing the time-invariant (trait-like) between-unit deviations from the grand means; and a within-component, captured by differences of the observed measurement at each time wave and its expected score, based on its grand mean and random intercept. In addition, several extensions of the model are possible, such as the inclusion of time-invariant predictors (Mulder and Hamaker, 2021).

The present study examined four models each (Models 1–4) for the use of the four mindfulness facets and their perceived helpfulness. A schematic graphical representation of the statistical models is presented in Figure 1. Model 1 allowed for autoregressive effects of all mindfulness facets and perceived stress from 1 day to the next, and the cross-lagged effects of all mindfulness facets to perceived stress on the following day, and of perceived stress to all mindfulness facets on the following day. In addition, the time-invariant predictors age, sex (0 = female, 1 = male), group (0 = community, 1 = student), SRA and OTE factor scores, baseline perceived stress, and meditation frequency were included as predictors of all daily observed measures.

Model 2 constrained the effects of the time-invariant predictors on the observed daily measures to be equal across waves, i.e., they were only used as predictors of the random intercepts [see Mulder and Hamaker (2021)]. Model 3 also constrained the autoregressive and cross-lagged effects, as well as the residual covariances, to be equal across time waves, to reduce model complexity. Lastly, Model 4 constrained the variances and covariances of the random intercepts to zero, yielding models that were statistically equivalent to the traditional CPLM, to allow comparisons of our results with this approach. Analyses were performed in R version 4.2.1, using the lavaan package version 0.6–14 (Rosseel, 2012).

#### 2.4.3 Data-analytic decisions

Across the entire set of study variables, 9.72% of observations were missing (i.e., 9,549 out of a total of 98,252 data points), and the percentage of missing observations was highest in the diary measures (11.56%; 9,289 of 80,388 cells). However, Little’s MCAR test (Little, 1988; computed with the R package naniar; Tierney et al., 2021) indicated that the probability of a missing observation was not associated with other cases of the same or other observed variables, *χ*^2^(10910) = 9,546, *p* = 1.00. Thus, missing values were handled in analysis via full-information maximum likelihood (FIML) estimation.

Concerning their distributions, scores in most measures and scales were approximately normally distributed, with some moderately skewed distributions (e.g., Nonjudge, meditation frequency). In the measurement and structural models, standard errors were thus estimated with robust maximum likelihood estimation (MLR), accounting for non-normality in endogenous variables.

Model fit was assessed by the comparative fit index (CFI) and the Tucker-Lewis index (TLI; good fit: >0.95, acceptable fit >0.90), the root mean square error of approximation (RMSEA; good fit: <0.05, acceptable fit <0.08), and the standardized root mean squared residual (SRMR; good fit: <0.08). Cutoffs were chosen according to Kenny (2020). To compare competing models, ΔCFI ≤0.010 (Cheung and Rensvold, 2002; Chen et al., 2008) and lower AIC and BIC values (Lin et al., 2017) were used as criteria. Satorra–Bentler *χ*^2^ tests are reported as well, but are not interpreted due to the large sample size (Kenny, 2020). We also note that while cutoff values for good and acceptable fit are a widely used alternative to the Satorra–Bentler *χ*^2^ test, they may depend on measurement and data conditions, making such global fit indices somewhat arbitrary as well (Barrett, 2007). The significance level was set to *α* = 0.05 (two-sided) in all analyses.

### 2.5 Open practices

Open data, open materials, and open code for this study are provided under https://osf.io/7tnwq/.

## 3 Results

Means, standard deviations, intercorrelations, and scale reliabilities (where applicable) for all time-invariant variables are displayed in Supplementary Table S1. The means and standard deviations of the time-varying, daily measured variables are reported in Supplementary Table S2.

### 3.1 Higher-order factors of mindfulness

The multigroup ESEM analysis indicated a good model fit, *χ*^2^(2) = 10.321, *p* = 0.006, CFI = 0.99, TLI = 0.897, RMSEA = 0.069 [0.019, 0.127], SRMR = 0.011. The low TLI value and relatively high RMSEA value could be attributed to the larger number of parameters (and, hence, low degrees of freedom) in this analysis, which is typical for ESEM models, were cross-loadings are estimated as well.

In both groups, Observe and Describe loaded highest on SRA, and Actaware, Nonjudge, and Nonreact loaded on OTE. In addition, in the community group, Nonreact loaded on SRA, and Describe on OTE. Descriptively, the association between SRA and OTE differed between the two groups, but it was not significant in either group (student group: *r* = 0.39, *p* = 0.149; community group: *r* = −0.08, *p* = 0.434). Factor loadings for both groups are displayed in Supplementary Figure S1.

### 3.2 Associations of the use of the mindfulness facets with daily stress

Models 1, 2, and 3 all had a good fit, and the differences in fit indices between the models were small and below the proposed cut-off values (see Table 2). Model 3 (constraining the effects of the time-invariant predictors, the autoregressive and the cross-lagged effects, and the residual variances all to be equal each across time points) proved to be the most parsimonious model and is reported in the following. Constraining the variances and covariances of the random intercepts to zero as well (Model 4, which resembled the traditional CPLM without random intercepts) resulted in a model with poor fit and large differences in the fit indices compared to the other models above the chosen cut-off values (see Table 2). We briefly describe the results from Model 4 in Section 3.4 below.

**Table 2:** Fit indices and model comparisons concerning the use and helpfulness of the mindfulness facets and daily stress.

									Model comparisons				
Model	*χ* ^2^	*df*	CFI	TLI	RMSEA	SRMR	AIC	BIC	∆*χ*^2^	∆*df*	∆CFI	∆AIC	∆BIC
Use of the mindfulness facets													
1	472.88	432	0.998	0.995	0.010	0.018	203109	205752					
2	713.54	642	0.996	0.995	0.010	0.023	202934	204495	240.66	210	−0.002	−175	−1257
3	854.49	755	0.995	0.994	0.011	0.025	202898	203877	140.95	113	−0.001	−36	−618
4	3205.55	770	0.869	0.854	0.054	0.116	205614	206515	2351.06	15	−0.126	2716	2638
Helpfulness of the mindfulness facets													
1	482.39	432	0.997	0.995	0.011	0.017	204880	207523					
2	693.44	642	0.997	0.997	0.009	0.020	204668	206229	211.05	210	<0.001	−212	−1294
3	817.91	755	0.997	0.996	0.009	0.022	204615	205594	124.47	113	<0.001	−53	−635
4	3115.05	770	0.882	0.868	0.054	0.140	207356	208257	2297.14	15	−0.115	2741	2663

#### 3.2.1 Within-subject effects

Detailed results are presented in Supplementary Table S3. The autoregressive paths from all time-varying variables to subsequent days were all significant (*p*s < 0.001). Standardized effect estimates were between 0.18 and 0.20 for Describe, between 0.19 and 0.21 for Actaware, between 0.21 and 0.22 for Nonjudge, between 0.23 and 0.24 for Nonreact, and between 0.28 and 0.30 for daily stress. Thus, daily stress appeared to have the largest carry-over effect to the following day.

Considering the cross-lagged effects, the more frequent use of Actaware predicted higher daily stress level on the following day (*β* = 0.03, *p* = 0.029), whereas the more frequent use of Nonreact predicted lower daily stress level on the following day (*β* = −0.04, *p* = 0.025). There were no cross-lagged effects for Describe and Nonjudge to perceived stress on the next day, or from perceived stress to any of the four mindfulness facets (*p* > 0.05). Further, there were significant correlations at t1 between Actaware and perceived stress (*r* = −0.10, *p* = 0.016) and between Nonreact and perceived stress (*r* = −0.25, *p* < 0.001). At t2–t7, there were residual correlations between Describe and daily stress (*r*s between −0.08 and −0.07, *p*s < 0.001) and between Nonreact and daily stress (*r*s between −0.26 and −0.21, *p*s < 0.001).

In summary, at the within-subject level, we found associations concerning the use of Actaware and Nonreact to perceived stress on the next day (partial support for hypothesis H1), but no effects from perceived stress to the use of mindfulness facets on the next day (no support for H2). In addition, we found covariation between the mindfulness facets and perceived stress within days.

#### 3.2.2 Between-subject effects

Detailed results are presented in Supplementary Table S4. The random intercepts of Describe and daily stress were positively correlated (*r* = 0.15, *p* = 0.003), indicating that participants who used the mindfulness facet Describe more frequently on average also reported higher daily stress on average. The random intercepts of Actaware, Nonjudge, and Nonreact were not associated with the random intercept of daily stress, but the random intercepts of all mindfulness facets intercorrelated positively with one another (*r*s ranging from 0.35 to 0.54, all *p*s < 0.001).

With regards to the covariates considered, women had higher random intercepts in Describe (*β* = −0.13, *p* < 0.001) and Nonjudge (*β* = −0.12, *p* < 0.001) than men, as was the case for younger vs. older participants (Describe: *β* = −0.13, *p* < 0.001; Nonjudge: *β* = −0.11, *p* = 0.010). Students had lower random intercepts in Actaware (*β* = −0.14, *p* < 0.001) and Describe (*β* = −0.07, *p* < 0.049) than members of the community, and meditation frequency was positively associated with the random intercepts in Nonjudge (*β* = 0.06, *p* = 0.045) and Nonreact (*β* = 0.07, *p* = 0.037).

The SRA and OTE were positively associated with the random intercepts of the mindfulness facets: SRA showed the highest relationship with the random intercepts in Describe (*β* = 0.23, *p* < 0.001) followed by Nonreact, Nonjudge and Actaware. OTE showed the highest relationship with the random intercepts of Actaware (*β* = 0.30, *p* < 0.001), followed by associations with Describe and Nonreact, but the relationship with Nonjudge was nominally not significant (*β* = 0.07, *p* = 0.074). Neither SRA (*β* = 0.01, *p* = 0.742) nor OTE (*β* = 0.06, *p* = 0.165) predicted the random intercepts of daily stress.

Baseline PSQ scores, in turn, were strongly positively associated with the random intercepts of daily stress (*β* = 0.61, *p* < 0.001). Interestingly, they were also negatively associated with the random intercepts of the mindfulness facets Actaware (*β* = −0.10, *p* = 0.011) and Nonreact (*β* = −0.16, *p* < 0.001).

In summary, at the between-subject level, we found associations of the use of the mindfulness facets with participant sex and age, student status, and meditation frequency. Only the random intercepts of Describe and perceived stress were correlated, and this association was positive (directionally opposed to the expectation of H3). However, higher baseline levels of stress predicted the less frequent use of the mindfulness facets Actaware and Nonjudge on average, but trait mindfulness did not predict the average level of daily stress.

### 3.3 Associations of the perceived helpfulness of the mindfulness facets with daily stress

As in the previous analysis, Model 3 emerged as the most parsimonious model (see Table 2). Again, the traditional CPLM (Model 4; see below) had only poor model fit.

#### 3.3.1 Within-subject effects

Detailed results are presented in Supplementary Table S5. Again, all autoregressive paths were significant (mindfulness facets: *β*s between 0.17 and 0.22; daily stress: *β* between 0.28 and 0.30; all *p*s < 0.001). Considering to the cross-lagged effects, higher perceived helpfulness of Nonreact predicted lower daily stress on the following day (*β* = −0.05, *p* = 0.014), but, in turn, higher daily stress predicted also lower perceived helpfulness of Describe (*β* = −0.04, *p* = 0.037) and Nonreact (*β* = −0.03, *p* = 0.038) on the following day. In addition, the perceived helpfulness of all mindfulness facets correlated with daily stress. These bidirectional effects provide partial support for hypotheses H1 and H2.

#### 3.3.2 Between-subject effects

Detailed results are presented in Supplementary Table S6. There were no associations of the random intercepts of the mindfulness facets with daily stress (again not supporting H3), but the random intercepts of all mindfulness facets intercorrelated positively with one another.

Again, women had higher random intercepts in Describe (*β* = −0.14, *p* < 0.001) and Nonjudge (*β* = −0.09, *p* = 0.004) than men. Older participants this time had *higher* random intercepts in Nonjudge (*β* = 0.13, *p* < 0.001) than younger participants. Meditation frequency was positively associated with the random intercepts of Describe (*β* = 0.06, *p* = 0.024), Nonjudge (*β* = 0.08, *p* = 0.006), and Nonreact (*β* = 0.10, *p* < 0.001).

SRA and OTE were, again, associated with the random intercepts of the mindfulness facets. The baseline PSQ scores were positively associated with the random intercepts of daily stress and negatively with the random intercepts of all mindfulness facets.

### 3.4 Results of the CPLM for comparison

#### 3.4.1 Use of mindfulness facets

The CPLM indicated cross-lagged effects that were not apparent in the above RI-CPLM: Daily stress positively predicted the more frequent use of Describe (*β* = 0.04, *p* < 0.001), Actaware (*β* = 0.04, *p* = 0.005), and Nonreact (*β* = 0.05, *p* < 0.001). In addition, daily stress and Nonreact correlated negatively at each time point (*r*s ranging from −0.14 to −0.22), and on t2–t7 daily stress also correlated negatively with the facets Describe (*r* = −0.06) and Actaware (*r* = −0.04; see Supplementary Table S7 for a full display of all parameter estimates).

#### 3.4.2 Helpfulness of the mindfulness facets

Again, the CPLM indicated cross-lagged effects that were not apparent in the above RI-CPLM: higher perceived helpfulness of Describe (*β* = 0.04, *p* = 0.025) and Nonjudge (*β* = 0.03, *p* = 0.026) predicted more daily stress on the subsequent day, and in reverse, more daily stress predicted higher perceived helpfulness of Describe (*β* = 0.04, *p* < 0.003), Actaware (*β* = 0.03, *p* = 0.014), and Nonreact (*β* = 0.03, *p* < 0.01) on the subsequent day. In addition, there were significant (residual) covariances between daily stress and all four mindfulness facets (see Supplementary Table S8 for a full display of all parameter estimates).

## 4 Discussion

The present study investigated the day-to-day associations between the use and perceived helpfulness of four mindfulness facets and perceived stress over the course of 1 week in a large, mixed student and community sample, using the random intercept cross-lagged panel model. We obtained evidence concerning the covariation of mindfulness and perceived stress on the same day, which is suggestive of construct overlaps reported for mindfulness and other mental-health-related outcomes on the trait (vs. state) level as well. However, we also observed a number of cross-lagged longitudinal associations. Longitudinal associations were (1) unidirectional from mindfulness to stress for Actaware (more frequent use predicted *more* subsequent stress), (2) bidirectional for Nonreact (more frequent use and perceived helpfulness predicted less subsequent stress and more prior stress predicted less perceived helpfulness), and (3) unidirectional from stress to mindfulness for Describe (more prior stress predicted less perceived helpfulness). The cross-lagged longitudinal associations were only small in magnitude and in part both corroborated and questioned prior results on the buffering and protective qualities of mindfulness against stress in daily life. Importantly, mindfulness also *increased* the amount of perceived stress in the present study, while stress appeared to interfere with the ability to stay mindful in daily life as well.

Beneficial effects of Nonreact on mental-health-related outcomes have already been highlighted in prior research. Nonreact was reported to facilitate adaptive emotion regulation strategies, like cognitive reappraisal, and prevent maladaptive emotion strategies, like suppression (Desrosiers et al., 2014; Curtiss et al., 2017). Additionally, Nonreact also loads on a common factor with decentering (Bednar et al., 2020), which is considered a core mechanism of stress regulation (Creswell and Lindsay, 2014).

According to the (extended) process model (e.g., Gross, 2015), emotion regulation operates in feedback loops in relation to situations that elicit emotional responses. Emotion regulation attempts to modify these emotional responses in five ways: situation selection and modification (altering the frequency or external aspects of emotional situations, e.g., via evasion); attentional deployment (directing attention toward or away from the emotional situation, e.g., via rumination or distraction); cognitive change (concerning the situation’s emotional meaning, e.g., trying to see positive aspects as well); and response modulation (changing the experiential, behavioral, and physiological responses themselves, e.g., via suppression of expression, the use of substances, exercise, or sleep).

One may argue that mindfulness more or less discourages options one to four and mainly focuses on option five, response modulation [cognitive change plays an important role in mindfulness as well, but likely is only an indirect consequence of an accepting and non-judgmental attitude rather than an explicit goal for most mindfulness practices; for a recent account on how mindfulness may affect emotion regulation, or may be considered an emotion-regulation strategy itself, see Raugh and Strauss (2023)]. Nonreact most closely corresponds to response modulation. Therefore, effects of Nonreact observed here and elsewhere may be grounded in its direct relation to emotion regulation, which is not equally the case for the other mindfulness facets [see Bednar et al. (2020)].

The present study thus suggests that Nonreact might not only have facilitating, or otherwise indirect, effects on mental health via its associations with emotion regulation (Desrosiers et al., 2014; Curtiss et al., 2017), but also direct effects on daily stress, because it may be considered an emotion regulation strategy by itself. In contrast, the other mindfulness facets might not have such effects, because they do not directly address emotion regulation [but see Raugh and Strauss (2023)]. This line of research should be followed up.

As only the use and perceived helpfulness of a distinct group of facets showed any longitudinal associations, hypotheses H1 and H2 were only partially supported. Also, the mindfulness facets had no uniform, and also not uniformly negative, effects on the day-to-day perception of stress (*cf*. Weinstein et al., 2009). Yet, the positive longitudinal association of Actaware with higher perceived stress was in line with previous results (Donald et al., 2016). Whether this association reflects a higher awareness of stress that actually is conducive to higher-quality self-regulation and coping (Donald et al., 2016) could not be directly supported. Coping mechanisms (apart from the mindfulness facets themselves) were not assessed in the present study. Also, the present study did not assess the actual *burden* of daily stress, but only its extent. Similarly paradoxical phenomena have been reported for the association between mindfulness and motivation in previous research as well. Motivation for tasks and goals, which did not align with one’s *own* values and interests, was reported to decrease with higher mindfulness, which could be equally considered beneficial rather than detrimental [see Walach et al. (2007) and Oberleiter et al. (2022)]. Yet, more research is currently still needed on such seemingly paradoxical effects of mindfulness.

Concerning the between-subject effects, there was only one relevant association between the random intercepts of the mindfulness facets and daily stress and this association was also *positive*; H3 was thus not supported. Participants who described their internal experiences with words more often also reported higher stress levels across the study period. This is again in line with findings that mindfulness may be associated not only with lower perceived stress, but also with *higher* stress.

The lack of further within-subject effects in the present study could have a methodological explanation as well. It is possible that there are lagged effects that just were not captured in our study design [see Rohrer and Murayama (2023), for an in-depth discussion on the interpretability of within-subject effects in the RI-CPLM]. Lagged effects could have occurred on shorter time scales, for example, from 1 h to the next, or, vice versa, effects could have been even more stable and could have occurred on even longer time scales, like from 1 week, or 1 month, to the next. Our data only captured a single week and were based on daily measurements. Future studies thus should investigate especially shorter time scales (i.e., collect more frequent real-time data over the course of each day) to rule out that associations were masked or blurred in the present study because of its design.

The observed residual covariances between the mindfulness facets and perceived stress on the same day could indeed be indicative of relevant shorter time scales that were not fully captured in our study design. At the same time, the observed associations of baseline perceived stress (PSQ scores) with the trait-like components (as captured in the random intercepts of the RI-CPLM) of the use of Actaware and Nonjudge indicated that longer time scales may have played some role as well. However, as Self-regulated Attention (SRA) and Orientation to Experience (OTE) did not likewise predict the average perceived stress level across the study period, these findings again appeared to favor a direction of stress to mindfulness regarding causality (i.e., stress interfering with the ability to stay mindful), rather than the other way around. Importantly, SRA and OTE did also predict the more frequent use and higher perceived helpfulness of the mindfulness facets across the study’s observation period, as did meditation experience. Still, this did not turn the observed order of associations around.

Staying mindful in daily life thus may also need to be considered a correlate or consequence of low prior stress rather than protecting against future stress. This finding fits nicely with prior meta-analytic results, which reported associations of changes in mindfulness with changes in mental health not only in mindfulness-based interventions, but also in non-mindfulness-based active control groups, and even in inactive control groups (Tran et al., 2022). It is also compatible with mounting evidence criticizing the construct validity of mindfulness (Goldberg et al., 2017) and highlighting its overlap with other constructs and mental health not only in empirical sample data (Bednar et al., 2020; Tran et al., 2020), but also in the item contents of widely used self-report scales themselves (Fischer et al., 2023).

Thus, the patterns observed in the present study may be possibly more or less independent of its temporal resolution. Instead, they might have to do with the way mindfulness is assessed, and is accessible, in self report. This topic is currently lively discussed in the literature [for an overview, see Burzler and Tran (2022)], but there may be no easy or immediate improvement or solution available. Probably, self-reported mindfulness cannot be sufficiently disentangled from other constructs, such as mental health or emotion regulation, at all.

Therefore, we also recommend utilizing non-self-report measures of mindfulness in future studies to rule out measurement bias and minimize common method variance. A number of behavioral measures have been proposed in the past [for an overview, see Treves et al. (2019)] and recently (e.g., Hadash et al., 2023). Alternatively, research may also switch to biological markers of stress and health [for overviews in the field of mindfulness intervention research, see Bossert et al. (2023) and Grasmann et al. (2023)]. However, many options might not easily lend themselves to the temporal resolution of the present study (or even shorter time scales). Some promising biomarkers of stress either operate only over longer periods of time [e.g., methylation of the serotonin transporter SLC6A4 gene; see Grasmann et al. (2023)] and/or might be too costly or complicated for (often-)repeated applications. Similarly, the behavioral mindful awareness task of Hadash et al. (2023) requires a 20-min mediation session to complete. Hence, not all problems may be adequately addressed via the use of such alternative approaches alone.

Concerning the FFMQ, we obtained further evidence for the good fit of a two-factor higher-order structure. This factor structure is thus not only theoretically in line with the two-component model of mindfulness (Bishop et al., 2004), but has up until now also been repeatedly empirically supported (in five independent datasets, including the current: Tran et al., 2013, 2014; Burzler and Tran, 2022; Borghi et al., 2023). In both the student and the community group, Observe and Describe loaded highest on SRA and Actaware, Nonjudge, and Nonreact on OTE. In the community group, Nonreact loaded on SRA as well. This pattern of results fits in nicely with previous reports.

In line with recent critique of the traditional CPLM (e.g., Hamaker et al., 2015; Lucas, 2023), modeling our data without random intercepts yielded a worse model fit and spurious cross-lagged effects (that were, however, also small in magnitude). We thus refrain from interpreting these additional observations as evidence for the presence of further such effects, but rather reported them to highlight the real possibility of statistical artifacts in the analysis of longitudinal data with inadequate methods.

Several effects of confounding variables on the use and perceived helpfulness of mindfulness were discernible. Women and younger participants reported more frequent use of the mindfulness facets Describe and Nonjudge than men and older participants and perceived those facets as more helpful as well. Participants, who mediated more often, also reported more frequent use of the mindfulness facets Nonjudge and Nonreact, and perceived the facets Describe, Nonjudge, and Nonreact as more helpful in the face of stress. In addition, students reported less frequent use of the mindfulness facet Actaware and Describe than non-students. This further highlights influences of student status, age, biological sex, and participants’ meditation experience and practice and, in turn, the necessity to consider basic demographic information and further person-level variables when studying the effects of mindfulness [see also Weinstein et al. (2009), Tran et al. (2013), and Burzler and Tran (2022)].

Summing up, we obtained evidence for both unidirectional and bidirectional associations between mindfulness and daily stress. Not all mindfulness facets appeared to contribute to stress-buffering effects, and stress may also interfere with the ability to stay mindful in daily life. The pattern of results is a likely consequence of *both* the conceptual ambiguity of mindfulness and its unique modus operandi. Future studies should strive to address issues relating to: (1) shorter time scales and, hence, high-frequency data collection over the course of the day; (2) the perceived burden of stress [and possibly also different components of stress, such as environmental, perceptual, or emotional; see Lobel and Dunkel-Schetter (1990)]; (3) alternative (i.e., non-self-report) measures of mindfulness and/or stress; (4) facilitating and indirect effects of mindfulness on further emotion regulation strategies; as well as (5) the actual malleability of the situations and events that are perceived as stressful by participants (because they might not be amenable to change at all – at least in the short run – which may further dilute the possible effects of mindfulness on daily stress).

### 4.1 Limitations

The present study also has some limitations. We used single-item measures for the daily measurements that conceivably are less reliable than multi-item inventories and did not assess the mindfulness facet Observe. The absence of Observe might have influenced the associations of the other facets with the investigated constructs. However, the two components of trait mindfulness, SRA and OTE, uniquely predicted the use and perceived helpfulness of mindfulness facets, and PSQ scores strongly predicted the average level of daily stress across the study period. This suggests that reductions in reliability or validity of the single-item measures were in all likelihood only small.

In addition, it may be that state mindfulness only protects from specific components of stress, such as environmental, perceptual or emotional components (Lobel and Dunkel-Schetter, 1990), for which the present stress measure did not differentiate. Potentially confounding variables, like neuroticism or emotion regulation, were not assessed. Given that only self-reports were used, the results may also be subject to common method variance. Further, the use and perceived helpfulness of the mindfulness facets, as well as daily stress, was assessed only retrospectively at the end of each day. Hence, reports could have been affected by recall bias and the momentary state at the time the diary was filled out. Also, as daily data were not collected in real-time (e.g., online), this could have introduced further undesirable sources of variance connected to recall bias. Finally, the temporal resolution of the present study (only daily measurements) may have masked or blurred relevant associations at shorter time scales.

Even though mindfulness and its beneficial effects are frequently discussed and studied in non-clinical populations, effects may be more noticeable in clinical populations (Keng et al., 2011). Accordingly, mindfulness may mitigate stress appraisals and reduce stress reactivity more noticeably only in high-stress populations (Creswell and Lindsay, 2014). We used a large (*N* > 1,000) sample from the general population, which has benefits in regards of generalizability and allowed for high statistical power. However, this comes at the cost of potentially overlooking effects that may become evident only in high-stress or clinical populations. Investigations of bidirectional day-to-day effects between mindfulness and stress in populations that may be specifically prone to high stress levels thus remain an important goal for future research as well.

## Data availability statement

The datasets presented in this study can be found in online repositories. The names of the repository/repositories and accession number(s) can be found at: https://osf.io/7tnwq/.

## Ethics statement

Ethical approval was not required for the studies involving humans because all procedures performed in this study adhere to the ethical standards of the 1964 Helsinki Declaration and its later amendments or comparable ethical standards. Study participation did not affect the physical or psychological integrity, the right for privacy, or other personal rights or interests of the participants. The studies were conducted in accordance with the local legislation and institutional requirements. The participants provided their written informed consent to participate in this study.

## Author contributions

OB: Conceptualization, Data curation, Formal analysis, Methodology, Visualization, Writing – original draft, Writing – review & editing. MV: Methodology, Writing – review & editing. UT: Conceptualization, Investigation, Methodology, Project administration, Supervision, Validation, Visualization, Writing – original draft, Writing – review & editing.
