# Supplementary material for: Day-to-day associations between mindfulness and perceived stress: insights from random intercept cross-lagged panel modeling
Source: Front Psychol. 2024 Apr 16;15:1272720. doi: 10.3389/fpsyg.2024.1272720 (PMC11062411; doi:10.3389/fpsyg.2024.1272720)
Supplement: Supplementary file 1 [file Data_Sheet_1.pdf]

Supplementary Material

Day-to-Day Associations between Mindfulness and Perceived Stress: Insights from Random Intercept Cross-Lagged Panel Modeling

Olaf Borghi[1], Martin Voracek[1,2], Ulrich S. Tran[1,2*]

[1] Department of Cognition, Emotion, and Methods in Psychology, Faculty of Psychology, University of Vienna, Vienna, Austria

[2] University Research Platform "The Stress of Life (SOLE) – Processes and Mechanisms underlying Everyday Life Stress", University of Vienna, Vienna, Austria

*** Correspondence:**
ulrich.tran@univie.ac.at

1 Supplementary Figures and Tables

1.1 Supplementary Figures

Figure S1
Two-Factor Higher-Order Structure of the FFMQ

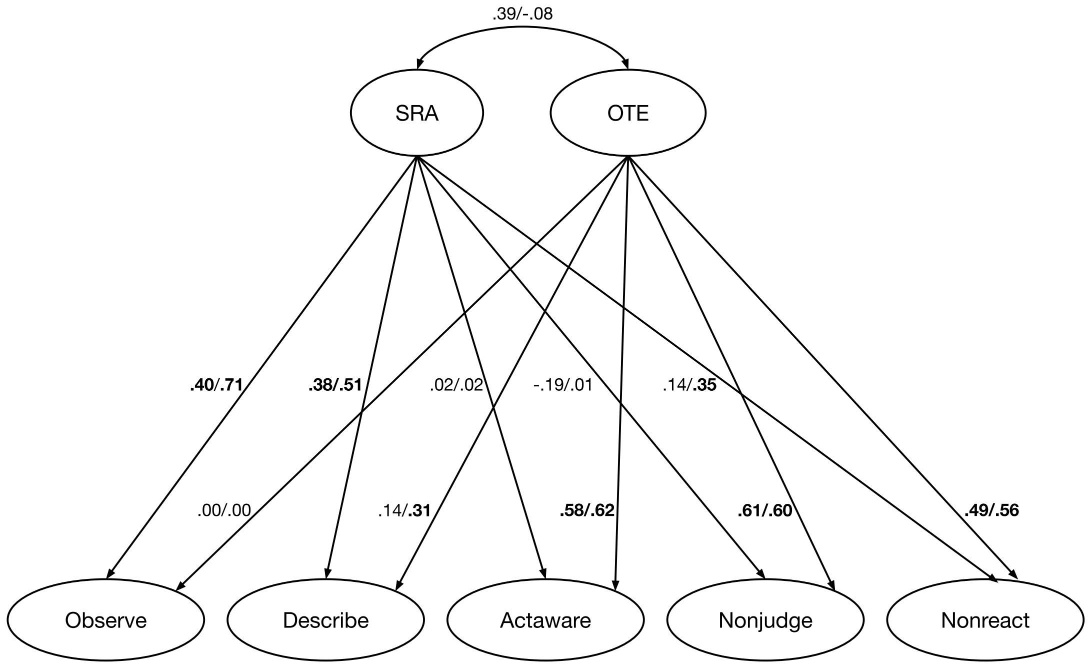

Note. Standardized factor loadings for students (left) and community (right) from multigroup ESEM analysis. Significant loadings (all *p*s < .01) are printed boldface, all other *p*s > .05. SRA = Self-regulated Attention; OTE = Orientation to Experience.

1.2 Supplementary Tables

Table S1

Means, Standard Deviations, Scale Reliabilities, and Intercorrelations of the Time-Invariant Variables.

Variable	*M*	*SD*	ω	1	2	3	4	5	6	7	8	9	10	11
1. Sex	1.47	0.50	-											
2. Age	32.37	14.68	-	-.02										
3. Student	1.50	0.50	-	.01	-.56**									
4. Meditation freq.	0.71	1.11	-	-.11**	.10**	-.06*								
5. Observe	14.55	3.05	.73	-.12**	.09**	-.08**	.27**							
6. Describe	14.42	3.02	.79	-.07**	.01	.01	.12**	.26**						
7. Actaware	13.16	3.12	.84	.08**	.17**	-.17**	.02	.04	.20**					
8. Nonjudge	15.07	3.41	.84	.05	.17**	-.08**	.06*	.01	.10**	.35**				
9. Nonreact	21.70	4.47	.83	.15**	.11**	-.03	.15**	.19**	.25**	.32**	.30**			
10. PSQ	40.04	19.07	.92	-.07*	-.13**	.07*	-.10**	-.12**	-.15**	-.36**	-.36**	-.34**		
12. SRA	0.00	0.69	-	-.08**	.05	.00	.26**	.81**	.67**	.15**	.01	.46**	-.18**	
11. OTE	0.00	0.78	-	.12**	.12**	.00	.08**	.06*	.36**	.76**	.72**	.69**	-.46**	.28**

Note. Meditation freq. = weekly frequency of meditation practice; PSQ = sum score of the Perceived Stress Questionnaire; SRA = Self-Regulated Attention; OTE = Orientation to Experience; ω = McDonald's ω (Cronbach's α values differed from these by .01 at most and were therefore omitted). Means, standard deviations, and correlations of OTE and SRA were calculated from the data.

* $p < .05$, ** $p < .01$.

Table S2

Means and Standard Deviations of the Daily Measured Use and Perceived Helpfulness of the Mindfulness Facets, and the Extent of Perceived Stress

Time point and	Use		Helpfulness	
variable	*M*	*SD*	*M*	*SD*
t1 Describe	7.07	2.56	5.84	2.99
t2 Describe	7.05	2.45	5.85	2.94
t3 Describe	7.08	2.45	5.81	2.96
t4 Describe	6.93	2.52	5.78	2.87
t5 Describe	6.95	2.45	5.80	2.85
t6 Describe	7.06	2.41	5.88	2.88
t7 Describe	7.07	2.52	5.85	2.98
t1 Actaware	6.60	2.57	6.25	2.97
t2 Actaware	6.50	2.56	6.18	2.92
t3 Actaware	6.48	2.60	6.20	2.85
t4 Actaware	6.46	2.48	6.23	2.82
t5 Actaware	6.46	2.52	6.12	2.86
t6 Actaware	6.46	2.56	6.19	2.85
t7 Actaware	6.47	2.59	6.11	2.90
t1 Nonjudge	6.01	2.82	5.03	3.01
t2 Nonjudge	6.06	2.71	5.05	2.96
t3 Nonjudge	5.97	2.76	5.04	2.95
t4 Nonjudge	5.78	2.76	5.02	2.86
t5 Nonjudge	5.90	2.68	5.05	2.90
t6 Nonjudge	5.89	2.72	5.03	2.94
t7 Nonjudge	5.85	2.74	5.05	2.94
t1 Nonreact	5.31	2.95	5.53	3.19
t2 Nonreact	5.37	2.85	5.39	3.06
t3 Nonreact	5.97	2.76	5.41	3.08
t4 Nonreact	5.34	2.83	5.27	3.03
t5 Nonreact	5.41	2.78	5.35	3.01
t6 Nonreact	5.54	2.80	5.48	3.04
t7 Nonreact	5.61	2.82	5.57	3.02
t1 Stress	4.13	2.52		
t2 Stress	4.26	2.47		
t3 Stress	4.36	2.52		
t4 Stress	4.40	2.46		
t5 Stress	4.46	2.45		
t6 Stress	4.35	2.52		
t7 Stress	4.16	2.61		

Note. For daily stress, the perceived extent was assessed, not use.

Table S3

Within-Subject Effects in Model 3 Concerning the Use of the Mindfulness Facets

Parameter	Est.	*SE*	*p*	95% *CI*	Std. est.
Auto-regressive paths					
**Describe**	**0.19**	**0.02**	**< .001**	**[0.15, 0.24]**	**[0.18, 0.20]**
**Actaware**	**0.20**	**0.02**	**< .001**	**[0.16, 0.23]**	**[0.19, 0.21]**
**Nonjudge**	**0.21**	**0.02**	**< .001**	**[0.17, 0.25]**	**[0.21, 0.22]**
**Nonreact**	**0.23**	**0.02**	**< .001**	**[0.19, 0.27]**	**[0.23, 0.24]**
**Stress**	**0.29**	**0.02**	**< .001**	**[0.25, 0.33]**	**[0.28, 0.30]**
Cross-lagged paths					
Describe → stress	-0.01	0.02	.532	[-0.05, 0.03]	-0.01
**Actaware → stress**	**0.04**	**0.02**	**.029**	**[0.004, 0.07]**	**0.03**
Nonjudge → stress	-0.002	0.02	.893	[-0.04, 0.03]	-0.002
**Nonreact → stress**	**-0.04**	**0.02**	**.025**	**[-0.08, -0.01]**	**-0.04**
Stress → Describe	-0.003	0.01	.825	[-0.02, 0.03]	0.004
Stress → Actaware	0.02	0.01	.186	[-0.01, 0.05]	0.02
Stress → Nonjudge	-0.02	0.01	.178	[-0.05, 0.01]	-0.02
Stress → Nonreact	-0.02	0.02	.186	[-0.05, 0.01]	-0.02
Covariances of daily measures at t1					
Stress with Describe	-0.19	0.13	.152	[-0.44, 0.07]	-0.05
**Stress with Actaware**	**-0.39**	**0.16**	**.016**	**[-0.70, -0.07]**	**-0.10**
Stress with Nonjudge	0.07	0.17	.686	[-0.26, 0.40]	0.02
**Stress with Nonreact**	**-1.09**	**0.17**	**< .001**	**[-1.43, -0.75]**	**-0.25**
Residual covariances of daily measures at t2-t7					
**Stress with Describe**	**-0.24**	**0.06**	**< .001**	**[-0.37, -0.12]**	**[-0.08, -0.07]**
Stress with Actaware	-0.14	0.09	.109	[-0.30, 0.03]	[-0.04, -0.03]
Stress with Nonjudge	0.01	0.08	.893	[-0.14, 0.16]	0.003
**Stress with Nonreact**	**-1.00**	**0.09**	**< .001**	**[-1.19, -0.81]**	**[-0.26, -0.21]**

Note. Est. = Unstandardized estimate (*b*); *SE* = standard error of the unstandardized estimate; 95% *CI* = 95% confidence interval of the unstandardized estimate; Std. est. = standardized estimate (β). The unstandardized (cross-)lagged parameters were constrained to be invariant across time, yet the standardized estimates could vary depending on the standard deviation of the variables at each time point. Wherever the difference between the minimum and maximum standardized estimate was > .01, its range is displayed. Significant parameters ($p < .05$) are printed boldface.

Table S4

Between-Subject Effects in Model 3 Concerning the Use of the Mindfulness Facets

Parameter	Est.	*SE*	*p*	95% *CI*	Std. est.
Covariances of random intercepts					
**Describe with Actaware**	**1.49**	**0.13**	**< .001**	**[1.24, 1.75]**	**0.54**
**Describe with Nonjudge**	**1.63**	**0.15**	**< .001**	**[1.34, 1.92]**	**0.46**
**Describe with Nonreact**	**1.16**	**0.14**	**< .001**	**[0.89, 1.44]**	**0.35**
**Actaware with Nonjudge**	**1.20**	**0.14**	**< .001**	**[0.93, 1.47]**	**0.40**
**Actaware with Nonreact**	**1.17**	**0.14**	**< .001**	**[0.90, 1.44]**	**0.42**
**Nonjudge with Nonreact**	**2.06**	**0.16**	**< .001**	**[1.75, 2.38]**	**0.58**
**Stress with Describe**	**0.30**	**0.10**	**.003**	**[0.10, 0.49]**	**0.15**
Stress with Actaware	0.11	0.10	.275	[-0.09, 0.30]	0.07
Stress with Nonjudge	0.03	0.12	.787	[-0.20, 0.26]	0.01
Stress with Nonreact	0.08	0.11	.493	[-0.14, 0.30]	0.04
Covariates predicting random intercepts					
Random intercept of Describe					
**Sex**	**-0.48**	**0.11**	**< .001**	**[-0.71, -0.26]**	**-0.13**
**Age**	**-0.02**	**0.005**	**.001**	**[-0.03, -0.01]**	**-0.13**
**Student status**	**-0.27**	**0.14**	**.049**	**[-0.54, -0.001]**	**-0.07**
Meditation frequency	-0.01	0.05	.877	[-0.10, 0.09]	-0.004
**SRA**	**0.64**	**0.10**	**< .001**	**[0.44, 0.84]**	**0.23**
**OTE**	**0.21**	**0.08**	**.013**	**[0.04, 0.38]**	**0.09**
PSQ	-0.01	0.004	.130	[-0.01, 0.002]	-0.05
Random intercept of Actaware					
Sex	-0.14	0.10	.186	[-0.34, 0.07]	-0.04
Age	-0.01	0.005	.088	[-0.02, 0.001]	-0.07
**Student status**	**-0.46**	**0.13**	**< .001**	**[-0.70, -0.21]**	**-0.14**
Meditation frequency	-0.06	0.04	.165	[-0.15, 0.03]	-0.04
**SRA**	**0.26**	**0.09**	**.003**	**[0.09, 0.43]**	**0.11**
**OTE**	**0.66**	**0.07**	**< .001**	**[0.51, 0.80]**	**0.30**
**PSQ**	**-0.01**	**0.003**	**.011**	**[-0.01, -0.002]**	**-0.10**
Random intercept of Nonjudge					
**Sex**	**-0.48**	**0.13**	**< .001**	**[-0.74, -0.23]**	**-0.12**
**Age**	**-0.01**	**0.01**	**0.01**	**[-0.03, -0.004]**	**-0.11**
Student status	-0.11	0.15	.466	[-0.41, 0.19]	-0.03
**Meditation frequency**	**0.11**	**0.05**	**.045**	**[0.003, 0.22]**	**0.06**
**SRA**	**0.39**	**0.11**	**< .001**	**[0.18, 0.60]**	**0.13**
OTE	0.18	0.10	.074	[-0.02, 0.37]	0.07
PSQ	-0.002	0.004	.650	[-0.01, 0.01]	-0.02
Random intercept of Nonreact					
Sex	-0.12	0.12	.348	[-0.36, 0.13]	-0.03
Age	-0.01	0.01	.264	[-0.02, 0.005]	-0.05
Student status	-0.11	0.15	.466	[-0.40, 0.18]	-0.03
**Meditation frequency**	**0.12**	**0.06**	**.037**	**[0.01, 0.23]**	**0.07**
**SRA**	**0.50**	**0.10**	**< .001**	**[0.29, 0.70]**	**0.17**
**OTE**	**0.37**	**0.09**	**< .001**	**[0.19, 0.55]**	**0.15**

**PSQ**	**-0.02**	**0.004**	**< .001**	**[-0.02, -0.01]**	**-0.16**
Random intercept of stress					
Sex	-0.06	0.09	.500	[-0.24, 0.11]	-0.02
Age	-0.01	0.004	.084	[-0.01, 0.001]	-0.07
Student status	-0.04	0.11	.715	[-0.25, 0.17]	-0.01
Meditation frequency	0.07	0.04	.095	[-0.01, 0.15]	0.06
SRA	0.02	0.07	.742	[-0.12, 0.16]	0.01
OTE	0.10	0.07	.165	[-0.04, 0.24]	0.06
**PSQ**	**0.04**	**0.003**	**< .001**	**[0.04, 0.05]**	**0.61**

Note. Est. = Unstandardized estimate (*b*); *SE* = standard error of the unstandardized estimate; 95% *CI* = 95% confidence interval of the unstandardized estimate; Std. est. = standardized estimate (β); SRA = Self-regulated Attention; OTE = Orientation to Experience; PSQ = sum score of the Perceived Stress Questionnaire.

Table S5

Within-Subject Effects in Model 3 Concerning the Helpfulness of the Mindfulness Facets

Parameter	Est.	*SE*	*p*	95% *CI*	Std. est.
Auto-regressive paths					
**Describe**	**0.21**	**0.02**	**< .001**	**[0.16, 0.25]**	**[0.20, 0.21]**
**Actaware**	**0.17**	**0.02**	**< .001**	**[0.13, 0.21]**	**[0.17, 0.19]**
**Nonjudge**	**0.20**	**0.02**	**< .001**	**[0.16, 0.24]**	**[0.20, 0.21]**
**Nonreact**	**0.20**	**0.02**	**< .001**	**[0.16, 0.24]**	**[0.19, 0.22]**
**Stress**	**0.29**	**0.02**	**< .001**	**[0.26, 0.33]**	**[0.28, 0.30]**
Cross-lagged paths					
Describe → stress	0.003	0.02	.883	[-0.04, 0.04]	0.003
Actaware → stress	0.004	0.02	.812	[-0.03, 0.03]	0.004
Nonjudge → stress	0.02	0.02	.263	[-0.02, 0.06]	0.02
**Nonreact → stress**	**-0.04**	**0.02**	**.014**	**[-0.08, -0.01]**	**-0.05**
**Stress → Describe**	**-0.03**	**0.02**	**.037**	**[-0.06, -0.002]**	**-0.04**
Stress → Actaware	< 0.001	0.02	.981	[-0.03, 0.03]	< 0.001
Stress → Nonjudge	-0.02	0.01	.117	[-0.05, 0.01]	-0.02
**Stress → Nonreact**	**-0.03**	**0.02**	**.038**	**[-0.07, -0.002]**	**-0.03**
Covariances of daily measures at t1					
**Stress with Describe**	**-0.84**	**0.17**	**< .001**	**[-1.16, -0.51]**	**-0.20**
Stress with Actaware	-0.20	0.18	.278	[-0.56, 0.16]	-0.04
**Stress with Nonjudge**	**-0.44**	**0.17**	**.008**	**[-0.77, -0.11]**	**-0.11**
**Stress with Nonreact**	**-0.89**	**0.19**	**< .001**	**[-1.25, -0.52]**	**-0.18**
Residual covariances of daily measures at t2-t7					
**Stress with Describe**	**-0.64**	**0.08**	**< .001**	**[-0.80, -0.49]**	**[-0.18, -0.16]**
**Stress with Actaware**	**-0.26**	**0.09**	**.002**	**[-0.43, -0.10]**	**-0.06**
**Stress with Nonjudge**	**-0.43**	**0.07**	**< .001**	**[-0.57, -0.28]**	**[-0.12, -0.11]**
**Stress with Nonreact**	**-0.90**	**0.10**	**< .001**	**[-1.09, -0.70]**	**[-0.22, -0.15]**

Note. Est. = Unstandardized estimate (*b*); *SE* = standard error of the unstandardized estimate; 95% *CI* = 95% confidence interval of the unstandardized estimate; Std. est. = standardized estimate (β). The unstandardized (cross-)lagged parameters were constrained to be invariant across time, yet the standardized estimates could vary depending on the standard deviation of the variables at each time point. Wherever the difference between the minimum and maximum standardized estimate was > .01, its range is displayed. Significant parameters ($p < .05$) are printed boldface.

Table S6

Between-Subject Effects in Model 3 Concerning the Helpfulness of the Mindfulness Facets

Parameter	Est.	*SE*	*p*	95% *CI*	Std. est.
Covariances of random intercepts					
**Describe with Actaware**	**2.35**	**0.17**	**< .001**	**[2.02, 2.68]**	**0.63**
**Describe with Nonjudge**	**2.83**	**0.18**	**< .001**	**[2.48, 3.17]**	**0.65**
**Describe with Nonreact**	**2.50**	**0.18**	**< .001**	**[2.14, 2.85]**	**0.59**
**Actaware with Nonjudge**	**1.96**	**0.16**	**< .001**	**[1.64, 2.28]**	**0.51**
**Actaware with Nonreact**	**1.92**	**0.17**	**< .001**	**[1.59, 2.25]**	**0.52**
**Nonjudge with Nonreact**	**2.71**	**0.18**	**< .001**	**[2.36, 3.07]**	**0.63**
Stress with Describe	0.18	0.12	.125	[-0.05, 0.41]	0.08
Stress with Actaware	0.21	0.11	.061	[-0.01, 0.43]	0.11
Stress with Nonjudge	0.08	0.12	.505	[-0.15, 0.31]	0.04
Stress with Nonreact	0.09	0.12	.452	[-0.15, 0.33]	0.04
Covariates predicting random intercepts					
Random intercept of Describe					
**Sex**	**-0.60**	**0.14**	**< .001**	**[-0.86, -0.34]**	**-0.14**
**Age**	**0.01**	**0.01**	**.015**	**[0.003, 0.02]**	**0.09**
Student status	-0.21	0.17	.198	[-0.54, 0.11]	-0.05
**Meditation frequency**	**0.13**	**0.06**	**.024**	**[0.02, 0.24]**	**0.06**
**SRA**	**0.54**	**0.11**	**< .001**	**[0.33, 0.75]**	**0.17**
**OTE**	**0.26**	**0.1**	**.014**	**[0.05, 0.46]**	**0.09**
**PSQ**	**-0.01**	**0.004**	**< .001**	**[-0.02, -0.01]**	**-0.12**
Random intercept of Actaware					
Sex	-0.18	0.12	.150	[-0.42, 0.06]	-0.05
Age	0.01	0.01	.223	[-0.004, 0.02]	0.05
Student status	-0.26	0.15	.078	[-0.55, 0.03]	-0.07
Meditation frequency	-0.03	0.05	.536	[-0.13, 0.07]	-0.02
**SRA**	**0.27**	**0.1**	**.009**	**[0.07, 0.47]**	**0.09**
**OTE**	**0.58**	**0.09**	**< .001**	**[0.40, 0.76]**	**0.23**
**PSQ**	**-0.01**	**0.004**	**.002**	**[-0.02, -0.004]**	**-0.12**
Random intercept of Nonjudge					
**Sex**	**-0.40**	**0.14**	**.004**	**[-0.67, -0.13]**	**-0.09**
**Age**	**0.02**	**0.01**	**< .001**	**[0.01, 0.03]**	**0.13**
Student status	-0.24	0.17	.168	[-0.57, 0.1]	-0.05
**Meditation frequency**	**0.17**	**0.06**	**.006**	**[0.05, 0.29]**	**0.08**
**SRA**	**0.46**	**0.11**	**< .001**	**[0.25, 0.68]**	**0.14**
**OTE**	**0.21**	**0.11**	**.047**	**[0.003, 0.42]**	**0.07**
**PSQ**	**-0.01**	**0.004**	**.016**	**[-0.02, -0.002]**	**-0.08**
Random intercept of Nonreact					
Sex	-0.27	0.14	.054	[-0.54, 0.004]	-0.06
Age	0.01	0.01	.123	[-0.003, 0.02]	0.06
Student status	-0.04	0.17	.807	[-0.37, 0.29]	-0.01
**Meditation frequency**	**0.20**	**0.06**	**.001**	**[0.08, 0.32]**	**0.10**
**SRA**	**0.45**	**0.11**	**< .001**	**[0.24, 0.66]**	**0.14**
**OTE**	**0.21**	**0.1**	**.037**	**[0.01, 0.41]**	**0.08**

**PSQ**	**-0.02**	**0.004**	**< .001**	**[-0.02, -0.01]**	**-0.14**
Random intercept of stress					
Sex	-0.06	0.09	.523	[-0.23, 0.12]	-0.02
Age	-0.01	0.004	.095	[-0.01, 0.001]	-0.07
Student status	-0.03	0.11	.751	[-0.25, 0.18]	-0.01
Meditation frequency	0.07	0.04	.099	[-0.01, 0.15]	0.06
SRA	0.03	0.07	.703	[-0.11, 0.17]	0.01
OTE	0.09	0.07	.201	[-0.05, 0.23]	0.05
**PSQ**	**0.04**	**0.003**	**< .001**	**[0.04, 0.05]**	**0.61**

Note. Est. = Unstandardized estimate (*b*); *SE* = standard error of the unstandardized estimate; 95% *CI* = 95% confidence interval of the unstandardized estimate; Std. est. = standardized estimate (β); SRA = Self-regulated Attention; OTE = Orientation to Experience; PSQ = sum score of the Perceived Stress Questionnaire.

Table S7

Within-Subject Effects in Model 4 (CPLM) Concerning the Use of the Mindfulness Facets

Parameter	Est.	*SE*	*p*	95% *CI*	Std. est.
Auto-regressive paths					
**Describe**	**0.61**	**0.01**	**< .001**	**[0.58, 0.64]**	**[0.60, 0.65]**
**Actaware**	**0.48**	**0.02**	**< .001**	**[0.45, 0.52]**	**[0.47, 0.51]**
**Nonjudge**	**0.59**	**0.01**	**< .001**	**[0.56, 0.62]**	**[0.57, 0.62]**
**Nonreact**	**0.54**	**0.02**	**< .001**	**[0.51, 0.57]**	**[0.54, 0.57]**
**Stress**	**0.44**	**0.01**	**< .001**	**[0.41, 0.47]**	**[0.42, 0.45]**
Cross-lagged paths					
Describe → stress	0.02	0.01	.096	[-0.004, 0.05]	0.02
Actaware → stress	0.02	0.01	.074	[-0.002, 0.05]	0.03
Nonjudge → stress	0.01	0.01	.626	[-0.02, 0.03]	0.01
Nonreact → stress	-0.01	0.01	.678	[-0.03, 0.02]	-0.01
**Stress → Describe**	**0.04**	**0.01**	**< .001**	**[0.02, 0.06]**	**0.04**
**Stress → Actaware**	**0.04**	**0.01**	**.005**	**[0.01, 0.06]**	**0.04**
Stress → Nonjudge	-0.01	0.01	.438	[-0.03, 0.02]	-0.01
**Stress → Nonreact**	**0.05**	**0.01**	**< .001**	**[0.03, 0.08]**	**0.05**
Covariances of daily measures at t1					
Stress with Describe	0.24	0.18	.187	[-0.11, 0.59]	0.04
Stress with Actaware	-0.11	0.19	.544	[-0.48, 0.25]	-0.02
Stress with Nonjudge	0.20	0.21	.337	[-0.21, 0.61]	0.03
**Stress with Nonreact**	**-0.94**	**0.21**	**< .001**	**[-1.36, -0.53]**	**-0.14**
Residual covariances of daily measures at t2-t7					
**Stress with Describe**	**-0.20**	**0.07**	**.005**	**[-0.34, -0.06]**	**-0.06**
**Stress with Actaware**	**-0.19**	**0.09**	**.034**	**[-0.37, -0.01]**	**-0.04**
Stress with Nonjudge	0.03	0.08	.715	[-0.14, 0.20]	0.01
**Stress with Nonreact**	**-0.96**	**0.10**	**< .001**	**[-1.16, -0.76]**	**[-0.22, -0.18]**

Note. Est. = Unstandardized estimate (*b*); *SE* = standard error of the unstandardized estimate; 95% *CI* = 95% confidence interval of the unstandardized estimate; Std. est. = standardized estimate (β). The unstandardized (cross-)lagged parameters were constrained to be invariant across time, yet the standardized estimates could vary depending on the standard deviation of the variables at each time point. Wherever the difference between the minimum and maximum standardized estimate was > .01, its range is displayed. Significant parameters ($p < .05$) are printed boldface.

Table S8

Within-Subject Effects in Model 4 (CPLM) Concerning the Helpfulness of the Mindfulness Facets

Parameter	Est.	*SE*	*p*	95% *CI*	Std. est.
Auto-regressive paths					
**Describe**	**0.58**	**0.01**	**< .001**	**[0.55, 0.61]**	**[0.58, 0.61]**
**Actaware**	**0.48**	**0.02**	**< .001**	**[0.45, 0.51]**	**[0.47, 0.50]**
**Nonjudge**	**0.58**	**0.01**	**< .001**	**[0.55, 0.61]**	**[0.57, 0.61]**
**Nonreact**	**0.53**	**0.02**	**< .001**	**[0.50, 0.56]**	**[0.51, 0.57]**
**Stress**	**0.45**	**0.01**	**< .001**	**[0.42, 0.47]**	**[0.43, 0.46]**
Cross-lagged paths					
**Describe → stress**	**0.03**	**0.01**	**.025**	**[0.004, 0.05]**	**0.04**
Actaware → stress	0.01	0.01	.240	[-0.01, 0.04]	0.02
**Nonjudge → stress**	**0.03**	**0.01**	**.026**	**[0.003, 0.05]**	**0.03**
Nonreact → stress	-0.01	0.01	.419	[-0.03, 0.01]	-0.01
**Stress → Describe**	**0.04**	**0.01**	**.003**	**[0.01, 0.07]**	**0.04**
**Stress → Actaware**	**0.04**	**0.01**	**.014**	**[0.01, 0.07]**	**0.03**
Stress → Nonjudge	0.02	0.01	.111	[-0.005, 0.05]	0.02
**Stress → Nonreact**	**0.04**	**0.01**	**.010**	**[0.01, 0.07]**	**0.03**
Covariances of daily measures at t1					
**Stress with Describe**	**-0.63**	**0.22**	**.004**	**[-1.06, -0.20]**	**-0.09**
Stress with Actaware	0.15	0.21	.473	[-0.27, 0.57]	0.02
Stress with Nonjudge	-0.40	0.21	.064	[-0.82, 0.02]	-0.06
**Stress with Nonreact**	**-0.75**	**0.23**	**.001**	**[-1.20, -0.30]**	**-0.10**
Residual covariances of daily measures at t2-t7					
**Stress with Describe**	**-0.67**	**0.09**	**< .001**	**[-0.84, -0.49]**	**-0.15**
**Stress with Actaware**	**-0.28**	**0.10**	**.003**	**[-0.46, -0.09]**	**-0.06**
**Stress with Nonjudge**	**-0.47**	**0.09**	**< .001**	**[-0.63, -0.30]**	**-0.10**
**Stress with Nonreact**	**-0.88**	**0.11**	**< .001**	**[-1.09, -0.67]**	**[-0.19, -0.13]**

Note. Est. = Unstandardized estimate (*b*); *SE* = standard error of the unstandardized estimate; 95% *CI* = 95% confidence interval of the unstandardized estimate; Std. est. = standardized estimate (β). The unstandardized (cross-)lagged parameters were constrained to be invariant across time, yet the standardized estimates could vary depending on the standard deviation of the variables at each time point. Wherever the difference between the minimum and maximum standardized estimate was > .01, its range is displayed. Significant parameters ($p < .05$) are printed boldface.
